# miR-675-5p enhances tumorigenesis and metastasis of esophageal squamous cell carcinoma by targeting REPS2

**DOI:** 10.18632/oncotarget.8950

**Published:** 2016-04-23

**Authors:** Yan-Wu Zhou, Hang Zhang, Chao-Jun Duan, Yang Gao, Yuan-Da Cheng, Dan He, Rong Li, Chun-Fang Zhang

**Affiliations:** ^1^ Department of Thoracic Surgery, Xiangya Hospital, Central South University, Changsha 410008, Hunan, China; ^2^ Institute of Medical Science, Xiangya Hospital, Central South University, Changsha 410008, Hunan, China; ^3^ Department of Gastroenterology, Xiangya Hospital, Central South University, Changsha 410008, Hunan, China

**Keywords:** miR-675-5p, REPS2, esophageal squamous cell carcinoma, microRNA, RAC1/CDC42

## Abstract

Recently H19 has been demonstrated to be up-regulated in esophageal squamous cell carcinoma (ESCC) and shown to be the precursor of miR-675 that encodes miR-675-5p conservatively. miR-675 is overexpressed in many human cancers; however, the function of miR-675-5p is largely unknown in ESCC. In this study, we found that miR-675-5p expression was significantly increased in ESCC tissues and cell lines and related with ESCC progression and poor prognosis. We also showed here that down-regulation of miR-675-5p in ESCC cells dramatically induced cell G1 arrest and reduced cell proliferation, colony formation, migration and invasion *in vitro* as well as tumorigenesis and tumor metastasis *in vivo*. We subsequently identified that REPS2 was a target gene of miR-675-5p. We found that inhibition of miR-675-5p up-regulated the expression of REPS2, inhibited RalBP1/RAC1/CDC42 signaling pathway. Inversely, interference of REPS2 abrogated the effect induced by miR-675-5p inhibition, which resembled the function of miR-675-5p up-regulation. Taken together, our findings suggested that miR-675-5p might play an oncogenic role in ESCC through RalBP1/RAC1/CDC42 signaling pathway by inhibiting REPS2 and might serve as a valuable prognostic biomarker and therapeutic target for ESCC patients.

## INTRODUCTION

Esophageal cancer occurs worldwide with a variable geographic distribution and ranks the seventh in order of incidence and the sixth as the leading cause of cancer mortality, affecting men more than women [1]. North-Central China is located in the highest risk “esophageal cancer belt” area, where the incidence rate can be as high as 800 cases per 100000 people and esophageal squamous cell carcinoma (ESCC) is the dominant histological type. The incidence and the mortality rates of ESCC have been steadily decreasing during the past several decades in China, on the contrary, it is continuing its march as the fastest growing malignancy in the western world [2]. Though the widespread application of radical esophagectomy and systemic chemoradiotherapy, ESCC patients still have a high mortality rate and poor prognosis due to the high prevalence of invasion and metastasis. Therefore, it is important for the control and treatment of this dreaded malignancy to identify and characterize clinically applicable tumor-specific molecular biomarkers for early detection and targeted prevention.

microRNAs (miRNAs) are a class of small (19–24 nucleotide) noncoding RNAs that mediate post-transcriptional regulation of target genes by translation repression or promoting RNA degradation and they are important in the regulation of various biological and pathological processes, such as cellular proliferation, differentiation, apoptosis and carcinogenesis. miRNAs are aberrantly expressed or mutated in a subset of human cancers, indicating that they may function as a novel species of oncogenes or tumor suppressor genes [3]. Although up to date multiple miRNAs and their molecular target genes have been implicated in the development of ESCC, the specific underlying mechanisms for their participation in the tumorigenesis and progression of ESCC are still unclear and need to be confirmed in clinical therapy.

H19 is a paternally imprinted oncofetal gene that encodes for a capped, spliced and polyadenylated 2.7 kb RNA [4, 5]. Recently H19 expression has been previously reported to be up-regulated in many cancers including hepatocellular carcinoma [6], ovarian cancer [7], bladder cancer [8], breast cancer [5], testicular cancer [9], choriocarcinoma [10], colorectal cancer [11], esophageal cancer [12], glioma [13], gastric cancer [14] and lung cancer [15]. Moreover, H19 has been shown to be the primary miRNA precursor of miR-675 in both human and mice [16]. It has been found that miR-675 is up-regulated in human colon cancer [17], serous endometrial tumors and endometrial carcinosarcomas [18], pancreatic cancer [19] and in blood cells of lung cancer patients compared to blood cells of COPD patients [20]. Overexpression of miR-675 also represents a potential new target for cartilage repair in osteoarthritis [21, 22]. However, other researchers argue that the H19 is a developmental reservoir of miR-675 that suppresses growth and Igf1r [23], meanwhile down-regulation of miR-675 is found in the group of adrenocortical carcinoma [24]. Interestingly, miR-675 is considered to be a disease remission–induced miRNA in patients with eosinophilic esophagitis [25]. H19 is overexpressed in ESCC tissues, we speculate that miR-675 might be involved in the tumorigenesis and progression of ESCC induced by H19. As one of the mature miRNAs of miR-675 in a classic Drosha and Dicer splicing dependent manner [16], miR-675-5p down-regulation was demonstrated to promote non-small cell lung carcinoma progression and development [26]. In this study, we found that miR-675-5p was overexpressed in ESCC tissues and cell lines compared to normal esophageal tissue and normal human HEEpic cell line and its expression was correlated with clinicopathological variables including lymph node metastasis, TNM stage, overall and disease-free survival of ESCC. miR-675-5p significantly promoted ESCC cell growth *in vitro* and *in vivo*. We further demonstrated that REPS2 (also known as POB1) was a direct target of miR-675-5p. miR-675-5p appeared to be a promising prognostic predictor and a potential therapeutic target in ESCC.

## RESULTS

### miR-675-5p was up-regulated in ESCC tissues and cell lines, positively correlated with H19 and associated with ESCC poor prognosis

**Figure 1:**
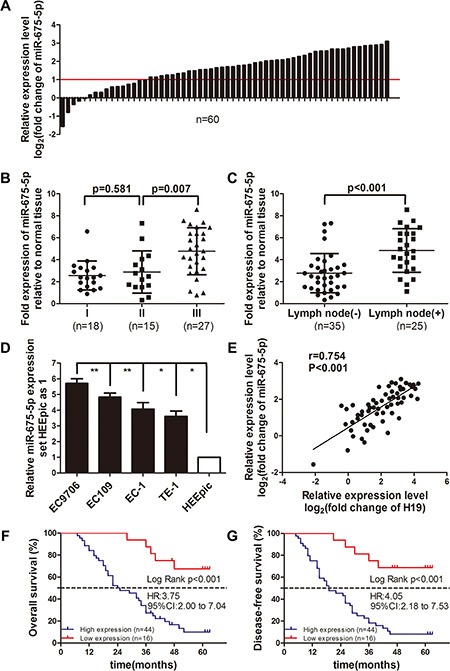
miR-675–5p was frequently up-regulated in ESCC, positively correlated with H19 and was a promising prognostic predictor for ESCC (**A**) Expression of miR-675-5p in 60 pairs of ESCC tissues and the adjacent normal esophageal tissues. Data were analyzed using a –ΔΔCT approach and expressed as log_2_fold change (−ΔΔCT). (**B**) Relative miR-675-5p expression levels in ESCC tissues at different TNM stages: I, II and III. (**C**) Relative miR-675-5p expression level in lymph node metastases: (+) or (−) ESCC tissues. (**D**) miR-675-5p expression in four ESCC cell lines and normal human esophageal epithelial cell line (HEEpic). Each sample was analyzed in triplicate and values were expressed as levels (mean ± SD) relative to those in HEEpic cells. (**E**) miR-675-5p expression was positively correlated with H19 mRNA in ESCC tissues. (**F**, **G**) Survival relevance analysis of miR-675-5p expression in ESCC patients. According to the qRT-PCR data, the expression of miR-675-5p was classified into high expression (*n* = 44) and low expression (*n* = 16). *P < 0.05, **P < 0.01.

microRNA-675 (miR-675) is overexpressed in many human cancers [5–10], however the function of miR-675-5p is largely unknown in ESCC. The expression of miR-675-5p was quantitatively analyzed by qRT-PCR in 60 pairs of primary ESCC and corresponding adjacent normal esophageal tissues. Results showed that the overall average expression level of miR-675-5p was markedly up-regulated (3.63 times) in tumor tissues than in adjacent normal tissues (Figure 1A). Comparative analysis of paired ESCC tissues with paired normal esophageal tissues further revealed that high miR-675-5p expression (more than 2-fold [i.e., log2 (fold change) > 1]) was observed in 73.3% cases (44/60), suggesting that overexpression of miR-675-5p was a frequent event in human ESCC (Figure 1A). We also found that miR-675-5p were overexpressed almost three fold in EC9706, Ec109, EC-1 and TE-1 cell lines compared with in HEEpic cell line (Figure 1D). Moreover, the expression of miR-675-5p was the highest in EC9706 cell line (Figure 1D). After that, we detected the expression of H19 in ESCC tissues and paired adjacent normal esophageal tissues. Our results verified that H19 was highly expressed in ESCC tissues and positively correlated with miR-675-5p (*r* = 0.754, *P* < 0.001, Figure 1E). Then we investigated the relevance of miR-675-5p expression and clinicopathological features. Data analysis showed that miR-675-5p high expression correlated with N Classification (*P* = 0.014) and TNM stage (*P* = 0.048) in ESCC (Table 1). Moreover, the miR-675-5p expression in advanced TNM stage (III) was higher than in early TNM stage (stage I or stage II) (Figure 1B). The miR-675-5p expression in lymph node metastases (+) (*n* = 25) ESCC tissues was significantly higher than in lymph node metastases (−) (*n* = 35) ESCC tissues (Figure 1C). However, miR-675-5p expression was not related to patients’ age, gender, drinking history, tumor differentiation, tumor size and T classification (Table 1). Hence, the initial results indicated that miR-675-5p was up-regulated in ESCC, suggesting that miR-675-5p might contribute to ESCC pathogenesis.

**Table 1:** Correlations between miR-675-5p expression level and clinicopathological variables of 60 cases of ESCC

		miR-675-5p	Expression	
Clinicopathological Variables	*n*	Low	High	*P*
Age (years)				
≤ 60	26	9	17	
> 60	34	7	27	0.252
Gender				
Female	8	2	6	
Male	52	14	38	1.000
Drinking history (years)				
≤ 10	11	3	8	
> 10	49	13	36	1.000
Differentiation				
Well	26	4	22	
Moderate	16	6	10	
Poor	18	6	12	0.216
Tumor size (cm)				
≤ 4	38	12	26	
> 4	22	4	18	0.367
T Classification				
T1	12	6	6	
T2	14	5	9	
T3	22	3	19	
T4	12	2	10	0.092
N Classification				
N0	35	14	21	
N1	12	2	10	
N2	13	0	13	**0.014**
TNM Stage				
I	18	7	11	
II	15	6	9	
III	27	3	24	**0.048**

To assess the feasibility of miR-675-5p expression in ESCC prognosis, the Cox proportional hazards regression model was introduced. On multivariate survival analysis, N classification (*P* = 0.042), TNM stage (*P* = 0.012) and miR-675-5p expression (*P* < 0.001) reached significance for overall survival (Table 2). Furthermore, ESCC patients with high miR-675-5p expression had much shorter overall survival time (median survival time, 24.5 versus more than 60 months, *P* < 0.001) than those with low miR-675-5p expression (Figure 1F). For analysis of disease-free survival time, N2 classification (*P* = 0.04), TNM stage (*P* = 0.013) and miR-675-5p expression (*P* < 0.001) reached significance in the multivariate survival analysis Cox proportional hazards regression model (Table 2). Similarly, ESCC patients with high miR-675-5p expression had shorter disease-free survival (median survival time, 19 versus more than 60 months, *P* < 0.001) than those with low miR-675-5p expression (Figure 1G).

**Table 2:** Cox regression multivariate analysis of overall and disease-free survival in 60 patients with ESCC

		Overall	Survival	Disease-Free	Survival
Variables	*n*	HR (95% CI)	*P*	HR (95% CI)	*P*
Age (years)					
≤ 60	26	1		1	
> 60	34	1.09 (0.40–2.94)	0.871	1.04 (0.41–2.67)	0.935
Gender					
Female	8	1		1	
Male	52	0.91 (0.30–2.81)	0.871	1.00 (0.32–3.10)	0.997
Drinking history (years)					
≤ 10	11	1		1	
> 10	49	0.93 (0.38–2.29)	0.877	0.87 (0.35–2.15)	0.768
Differentiation			0.392		0.307
Well	26	1		1	
Moderate	16	1.28 (0.40–4.16)	0.680	1.07 (0.33–3.44)	0.915
Poor	18	0.62 (0.25–1.58)	0.320	0.53 (0.21–1.34)	0.179
Tumor size (cm)					
≤ 4	38	1		1	
> 4	22	0.53 (0.19–1.51)	0.235	0.56 (0.20–1.55)	0.262
T Classification			0.073		0.132
T1	12	1		1	
T2	14	1.17 (0.23–5.92)	0.852	1.28 (0.28–5.78)	0.747
T3	22	1.13 (0.27–4.74)	0.871	1.01 (0.26–3.89)	0.989
T4	12	5.06 (0.89–28.67)	0.067	3.63 (0.68–19.48)	0.132
N Classification			**0.042**		0.091
N0	35	1		1	
N1	12	2.65 (0.52–13.38)	0.240	2.15 (0.43–10.64)	0.350
N2	13	8.82 (1.50–51.88)	**0.016**	5.75 (1.08–30.59)	**0.040**
TNM Stage			**0.012**		**0.006**
I	18	1		1	
II	15	7.55 (1.60–35.74)	**0.011**	6.30 (1.48–26.90)	**0.013**
III	27	16.80 (2.28–123.78)	**0.006**	22.43 (3.19–157.92)	**0.002**
miR-675-5p expression					
Low	16	1		1	
High	44	10.32 (2.97–35.89)	**< 0.001**	14.32 (3.88–52.82)	**< 0.001**

### Down-regulation of miR-675-5p induced cell G1 arrest, reduced cell proliferation, colony formation, migration, invasion, tumorigenicity and tumor metastasis

**Figure 2:**
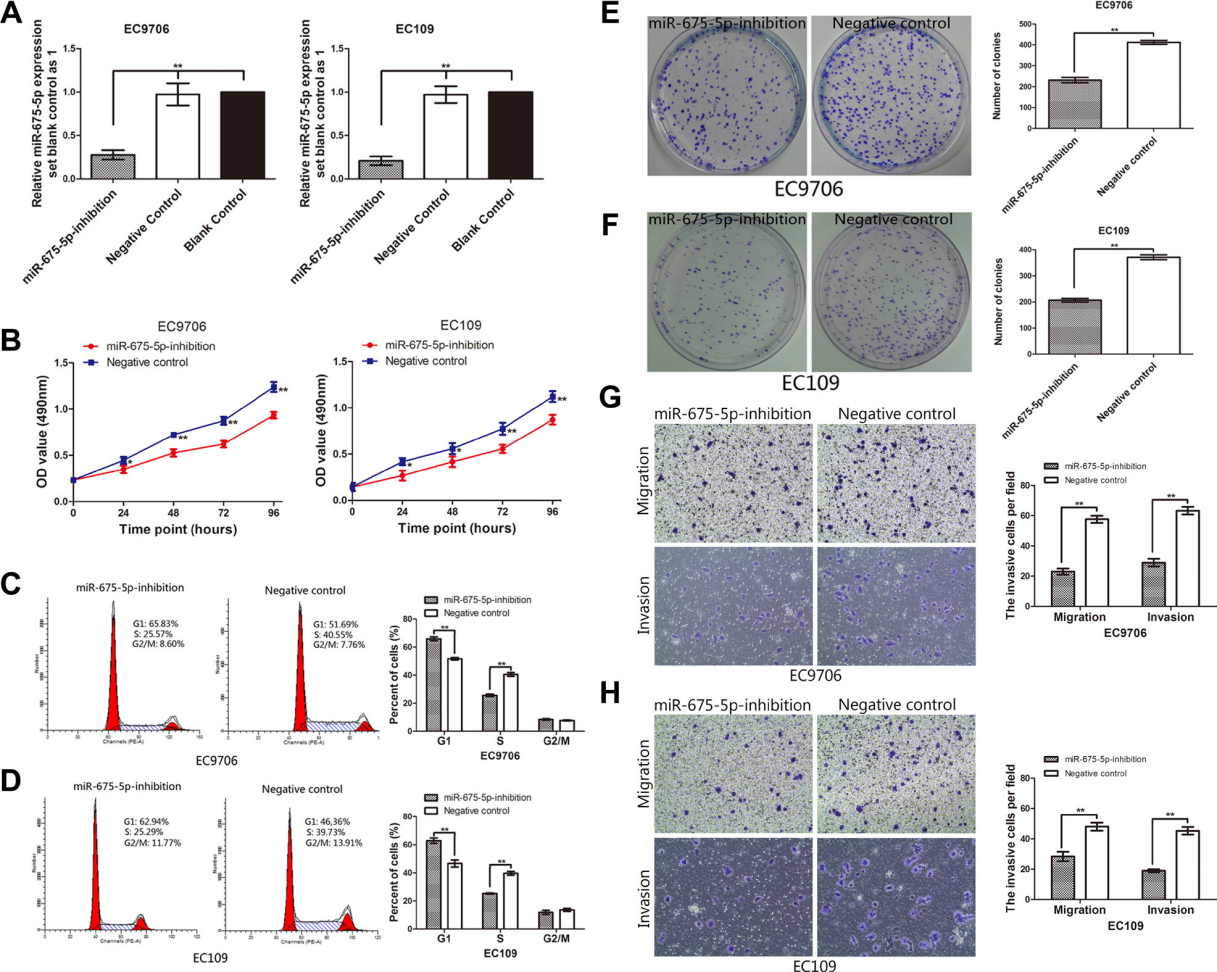
Inhibition of miR-675-5p induced cell G1 arrest, reduced proliferation, colony formation, *vitro* migration and invasion of ESCC cells (**A**) The level of miR-675-5p in EC9706 and EC109 cells was significantly down-regulated after transfection with LV-miR-675-5p-inhibition. (**B**) Down-regulation of miR-675-5p reduced cell proliferation in ESCC cells. Cell proliferation was determined by MTT assays. (**C**, **D**) Down-regulation of miR-675-5p induced cell cycle arrest at the G1/S phase. (**E**, **F**) Down-regulation of miR-675-5p suppressed colony formation compared with negative control (namely cells transfected with LV-miR-675-5p-NC). The number of colonies were calculated and depicted by the ban graph. (**G**, **H**) The number of migrating or invading cells in the miR-675-5p-inhibition group was significantly decreased compared with the negative control group (namely cells transfected with LV-miR-675-5p-NC). Data were represented as the mean ± SD of three independent experiments. *P < 0.05, **P < 0.01.

In current studies, in order to investigate the potential impact of miR-675-5p on proliferation, apoptosis, colony formation, migration, invasion, tumorigenicity and tumor metastasis of ESCC cells, we transfected EC109 and EC9706 cells which had high basal levels of miR-675-5p in ESCC cell lines with LV-miR-675-5p-inhibition (named miR-675-5p-inhibition EC109 or EC9706 cells) or LV-miR-675-5p-NC (named negative control cells). As shown in Figure 2A, both in EC109 and EC9706 cells, miR-675-5p was significantly inhibited in miR-675-inhibition group compared with negative control and blank control group (cells without any treatment); however, no significant difference was found between negative control and blank control group. Consequently, we selected miR-675-inhibition group and negative control group for the further analysis.

In order to investigate the impact of miR-675-5p on cell proliferation and cell cycle progress, MTT assay and flow cytometry were conducted. The data showed that down-regulation of miR-675-5p suppressed the proliferation of EC9706 and EC109 cells (Figure 2B). Similarly, colony formation assays showed that cell proliferation in both EC9706 and EC109 cells was significantly repressed by down-regulation of miR-675-5p (Figure 2E, 2F).

To explore the possible mechanism underlying the inhibitory effect on cell growth by down-regulation of miR-675-5p, cell cycle analysis was performed. The data showed that down-regulation of miR-675-5p inhibited cell cycle by inducing G1 arrest and decreased the percentages of EC9706 and EC109 cells in S phase compared to the negative control (Figure 2C and 2D). Whereas there was no significant difference of apoptotic rate between the cells transfected with LV-miR-675-5p-inhibition and control cells (*P >* 0.05) by Annexin V fluorescein isothiocyanate (V-FITC) apoptotic assay (data not shown).

Transwell migration and matrigel invasion assays were performed to assess the effect of miR-675-5p on cell migration and invasion. As shown in Figure 2G and Figure 2H, compared to the negative control, down-regulation of miR-675-5p could effectively repress the migration ability and invasion capacity of EC9706 and EC109 cells, respectively, indicating oncogenic role of miR-675-5p on the migration and invasion of ESCC.

**Figure 3:**
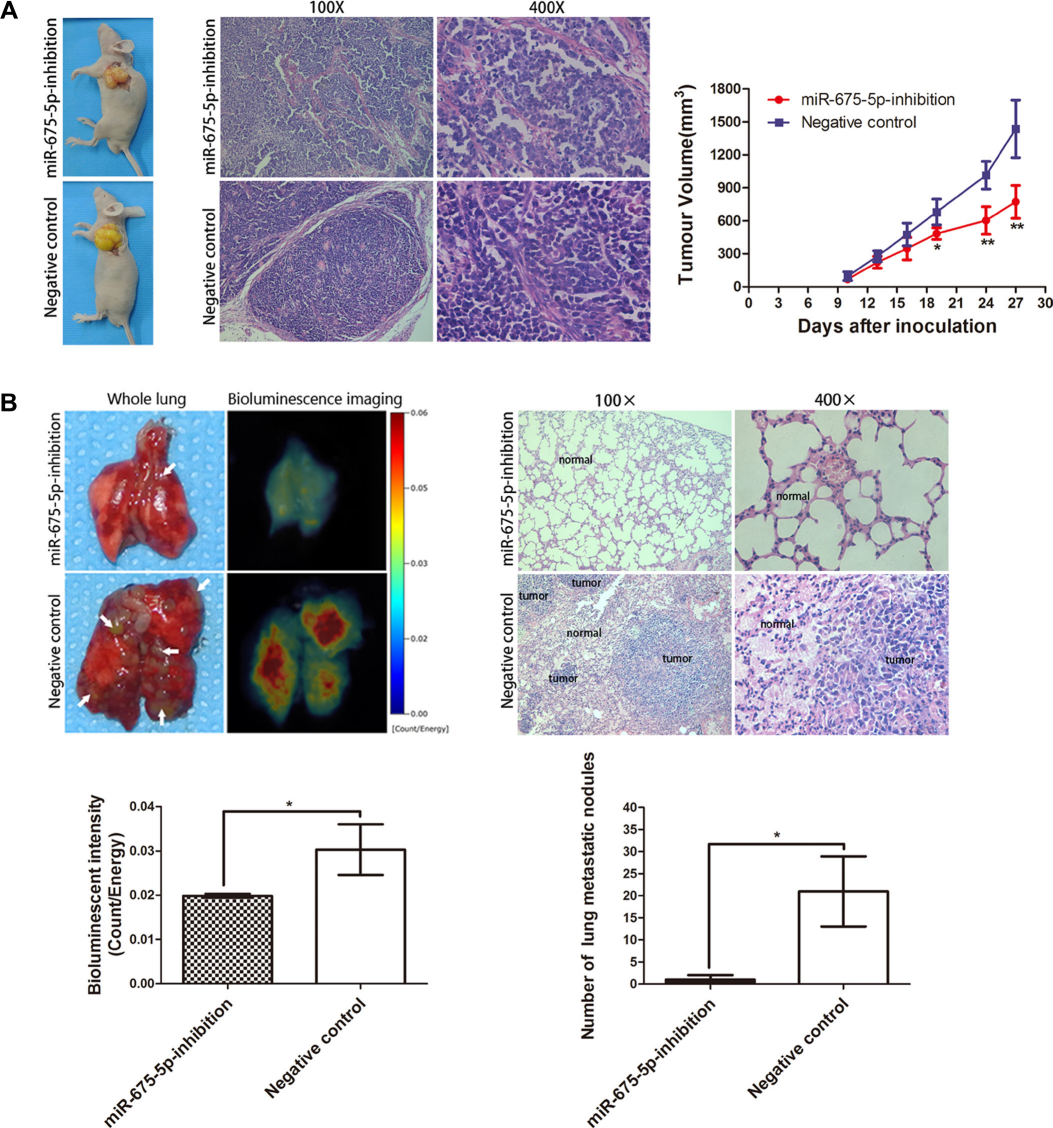
Inhibition of miR-675-5p inhibited *vivo* tumorigenicity and tumor metastasis of ESCC cells (**A**) Representative picture of ESCC subcutaneous implantation models (left), HE staining of the tumor (middle), tumor volumes and tumor growth curves (right) of each group are shown. (**B**) Representative picture and the bioluminescence image of murine whole lung on 35th day are presented (upper left). The bioluminescent change in miR-675-5p-inhibition group was significantly decreased compared with the negative control (namely cells transfected with LV-miR-675-5p-NC) (upper left and lower left). The number of visible surface metastatic lesions in miR-675-5p group was greatly decreased than in the negative control group (namely cells transfected with LV-miR-675-5p-NC) (lower right) and HE staining of the lung was presented (upper left). *P < 0.05, **P < 0.01.

To further explore the role of miR-675-5p on tumorigenicity and tumor metastasis *in vivo*, miR-675-5p-inhibition EC9706 cells and negative control cells were inoculated into the left upper flank region of nude mice or into the tail veins of nude mice. For subcutaneous tumor formation, tumor growth was evaluated for 28 days after injection. The tumor growth-curve of tumor volume was drawn according to time and significant difference was shown between the two groups (Figure 3A, right). Greater tumors were detected in mice injected with negative control cells compared with those injected with miR-675-5p-inhibition EC9706 cells (Figure 3A, left and right). The size of ESCC tumors in these two groups was calculated and compared. The average tumor volume of miR-675-5p-inhibition group was 772.97 ± 149.44 mm^3^, which was significantly smaller than tumors in the negative control group (1436.83 ± 261.45 mm^3^) (Figure 3A, right, on 28th day). The HE staining of tumor in each group was also showed (Figure 3A, middle). And for tumor metastasis analysis, lung metastatic tumors were apparent in mice injected with negative control cells via tail veins injection. In contrast, few metastatic tumors were detected in mice injected with miR-675-5p-inhibition EC9706 cells (Figure 3B, upper left and lower right). Based on bioluminescence imaging, down-regulation of miR-675-5p inhibited tumorigenic ability compared with those in negative control cells injected mice (Figure 3B, upper left and lower left). The histological examination of lung tissue indicated that the number of lung metastatic nodules significantly decreased in mice inoculated with miR-675-5p-inhibition EC9706 cells compared to the negative control (Figure 3B, upper right). In short, our results indicated that decreased expression of miR-675-5p inhibited ESCC cell tumorigenicity and tumor metastasis *in vivo*.

### REPS2 was a direct target gene of miR-675-5p

**Figure 4:**
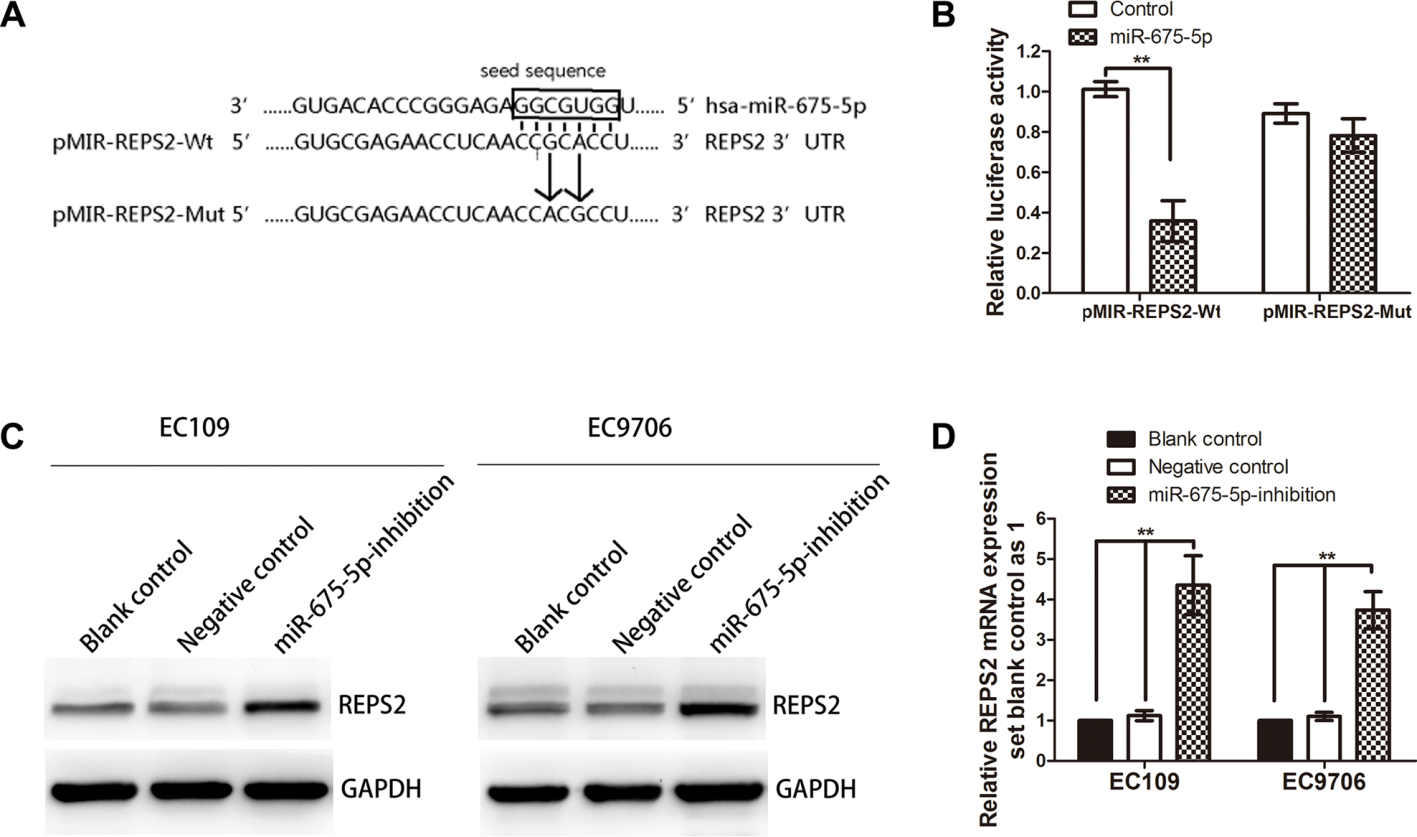
REPS2 was a direct downstream target of miR-675-5p (**A**) Schematic of the construction of wild-type or mutant pMIR-REPS2 3′-UTR vectors was illustrated. (**B**) Relative luciferase activity was analyzed in EC9706 cells. Firefly luciferase vector was used as an internal control. (**C**, **D**) Related expression of REPS2 protein and mRNA in EC109 and EC9706 cells treated with LV-miR-675-5p-inhibition or negative control (namely cells transfected with LV-miR-675-5p-NC) or blank control (namely cells without any treatment) was determined by western blotting analysis and qRT-PCR analysis. Data were represented as the mean ± SD of three independent experiments. *P < 0.05, **P < 0.01.

In order to disclose the molecular mechanism through which miR-675-5p realizes its tumor-stimulative functions, computational prediction using open access websites including miRDB, DIANA-MICROT, MICRORNA.ORG and TargetScan found that REPS2 was a strong potential target. miRDB predicted that the 3′UTR of REPS2 mRNA contained a complementary site for the seed region of miR-675-5p (Figure 4A). What is more, REPS2 plays important roles in inhibiting cell proliferation and migration of cancer cells [27–29]. Therefore, REPS2 was selected for further analysis. To confirm whether REPS2 was a direct target of miR-675-5p, a dual-luciferase reporter system was used, employing cotransfection of miR-675-5p and a luciferase reporter plasmid containing the 3′UTR of human REPS2. As shown in Figure 4B, the intensity of fluorescence was reduced significantly after miR-675-5p mimic cotransfection compared with the negative control. However, no significant variation in luciferase activity was observed for either the REPS2-Mut or the negative control miR-675-5p cotransfection. Thus, the luciferase assays revealed that miR-675-5p could bind to the REPS2 3′UTR, causing a significant decrease in luciferase activity compared with the negative control.

Furthermore, western blotting analysis showed that REPS2 protein expression was greatly up-regulated in EC9706 and EC109 cells after LV-miR-675-5p-inhibition transfection compared with the negative control (Figure 4C). In addition, qRT-PCR analysis showed that REPS2 mRNA apparently increased after LV-miR-675-5p-inhibition transfection in EC9706 and EC109 cell lines (Figure 4D). These data suggested that REPS2 expression was negatively regulated by miR-675-5p.

In our previous study, we found that REPS2 mRNA and protein expression levels were down-regulated in ESCC tissues and cell lines [30]. These data indicated that REPS2 was a direct target of miR-675-5p.

### Down-regulation of REPS2 by REPS2 interference dramatically reduced cell G1 arrest and induced cell proliferation, colony formation, migration and invasion, which mimicked the effect of miR-675-5p up-regulation

**Figure 5:**
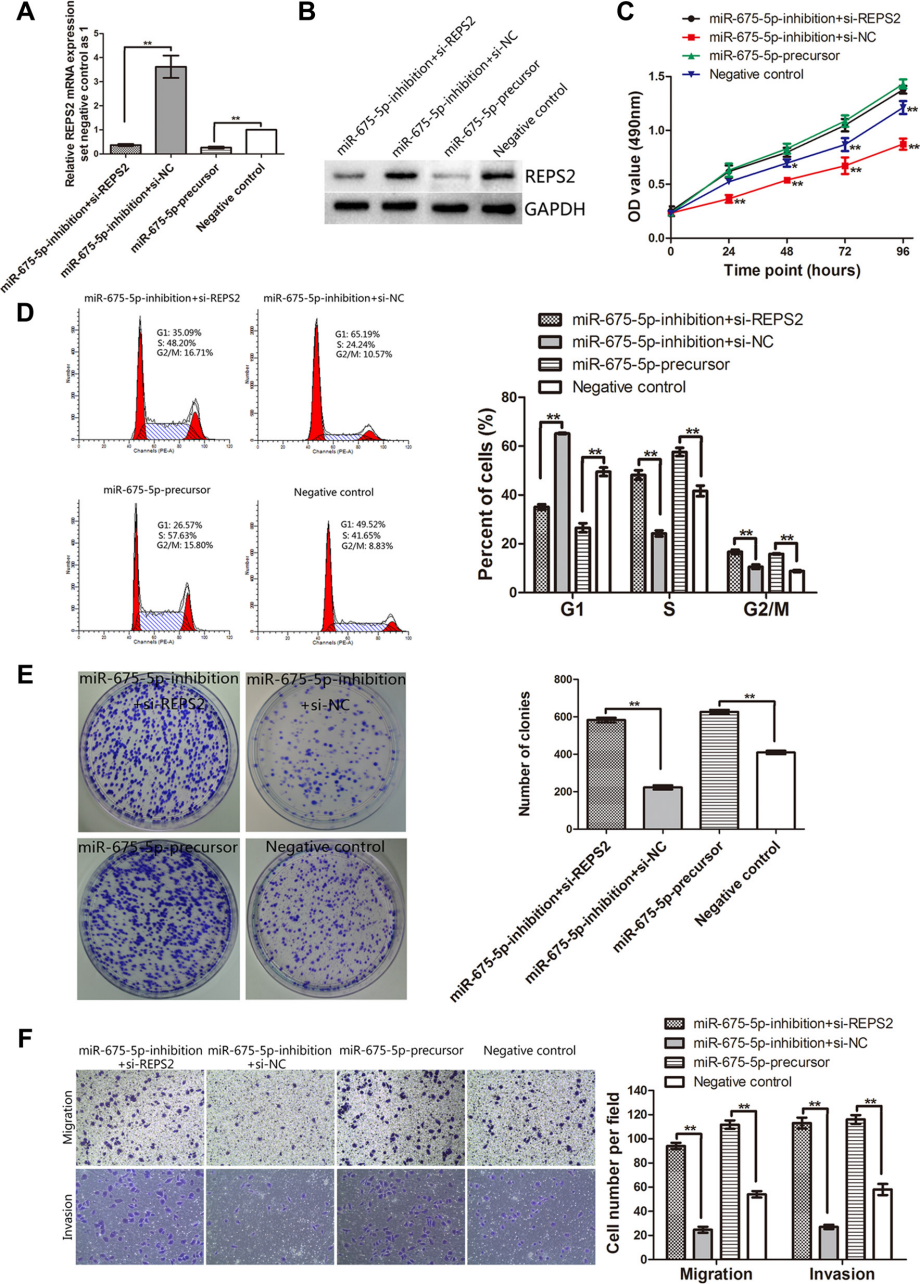
Interference of REPS2 RNA in miR-675-5p-inhibition EC9706 cells mimicked the oncogenic function of miR-675-5p up-regulation in EC9706 cells (**A**, **B**) The up-regulated REPS2 mRNA and protein in miR-675-5p-inhibition EC9706 cells was significantly down-regulated after transfection with si-REPS2, which mimicked the effect of miR-675-5p up-regulation. Knockdown of REPS2 significantly induced proliferation (**C**), reduced cell G1 arrest (**D**), promoted colony formation (**E**), migration and invasion (**F**) in miR-675-5p-inhibition EC9706 cells, which was similar to the function of up-regulating miR-675-5p in EC9706 cells. *P < 0.05, **P < 0.01.

Our above study had demonstrated that EC9706 cells transfected with LV-miR-675-5p-inhibition displayed higher expression of REPS2 mRNA and protein and exhibited lower cell proliferation, colony formation, migration and invasion potential when compared with the negative control (Figure 2B–2H, Figure 4C, 4D). To investigate whether miR-675-5p exerts its oncogenic function by targeting REPS2, we examined whether knockdown of REPS2 expression by REPS2 interference could recover the oncogenic effects of miR-675-5p in miR-675-5p-inhibition EC9706 cells. And we also observed the effect of REPS2 inhibition by up-regulating miR-675-5p (named miR-675-5p-precursor). The miR-675-5p-inhibition EC9706 cells group transfected with si-REPS2 (named miR-675-5p-inhibition+si-REPS2) or the miR-675-5p-precursor group displayed lower expression of REPS2 mRNA and protein compared to the negative control group (named miR-675-5p-inhibition+si-NC or negative control, respectively) (Figure 5A and 5B). Interestingly, the miR-675-5p-inhibition+si-REPS2 EC9706 cells exhibited higher cell proliferation, colony formation, migration and invasion potential and lower cell G1 arrest when compared with the miR-675-5p-inhibition+si-NC EC9706 cells, which resembled the miR-675-5p-precursor EC9706 cells (Figure 5C, 5D, 5E, 5F). Whereas there was no significant difference of apoptotic rate between these groups (*P >* 0.05) by Annexin V fluorescein isothiocyanate (V-FITC) apoptotic assay (data not shown). These results indicated that reduction of REPS2 expression could promote EC9706 cells to proliferate, migrate and invade. miR-675-5p acted as an oncogenic miRNA in EC9706 cells by down-regulating REPS2 expression.

### miR-675-5p promoted the RalBP1/RAC1/CDC42 signaling pathway by inhibiting REPS2

**Figure 6:**
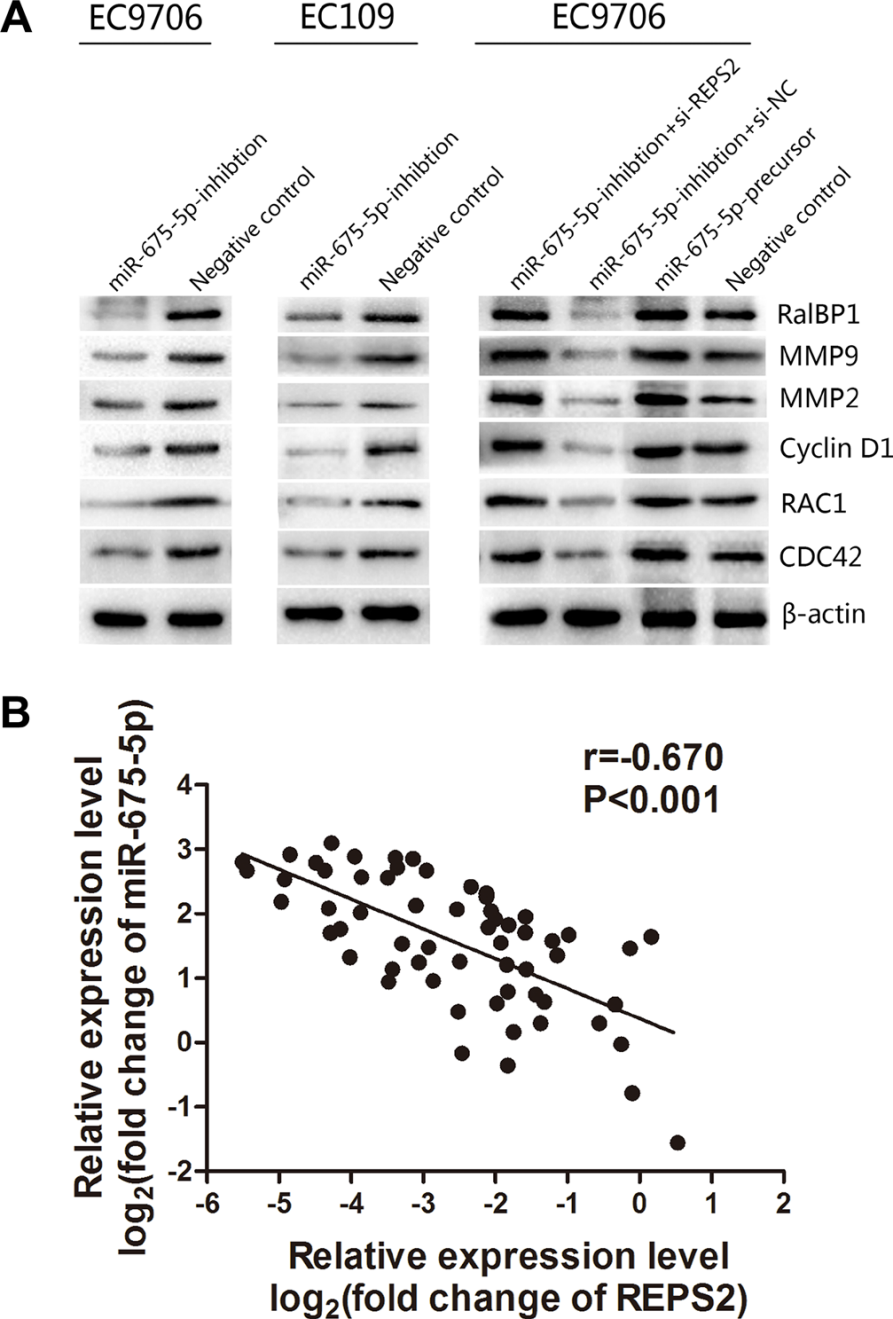
miR-675-5p mediated the promotion of the RalBP1/RAC1/CDC42 signaling pathway by regulating REPS2 and inversely correlated with the expression of REPS2 (**A**) In EC9706 and EC109 cells with miR-675-5p down-regulation, the protein levels of RalBP1, RAC1, CDC42, MMP9, MMP2 and Cyclin D1 were significantly decreased compared with the negative control (namely cells transfected with LV-miR-675-5p-NC), whereas these decreased proteins in miR-675-5p-inhibition EC9706 cells were significantly increased when treated with si-REPS2 compared to the negative control (namely miR-675-5p-inhibition+si-NC), which resembled the effect of miR-675-5p up-regulation in EC9706 cells. (**B**) miR-675-5p was negatively correlated with REPS2 mRNA in the ESCC samples.

To explore whether miR-675-5p exerts its functions through the REPS2/RalBP1/RAC1/CDC42 signaling pathways that contribute to cancer proliferation, development and progression [27–29, 31], we examined a number of the main REPS2 signaling downstream target genes, including RalBP1 which possessed GAPase activity, RAC1 and CDC42 with GTPase activity, MMP9 and MMP2 which were demonstrated to be associated with tumor dissemination and metastasis and Cyclin D1 correlated with G1-S phase transition. Expression of RalBP1, RAC1, CDC42 MMP9, MMP2 and CyclinD1 were decreased in miR-675-5p-inhibition EC9706 and EC109 cells (Figure 6A, left and middle, lane 1). Moreover, knockdown of REPS2 expression in miR-675-5p-inhibition EC9706 cells by si-REPS2 (Figure 6A, right, lane 1) abrogated the effects induced by miR-675-5p down-regulation (Figure 6A, left, lane 1), which was similar to the effect of miR-675-5p up-regulation in EC9706 cells (Figure 6A, right, lane 3). These data indicated that miR-675-5p promoted RalBP1/RAC1/CDC42 signaling by inhibiting REPS2 in ESCC, which was involved in tumor development and progression.

### miR-675-5p expression inversely correlated with the expression of REPS2 in ESCC

In our previous study, we found that REPS2 mRNA and protein expression levels were down-regulated in ESCC tissues and cell lines [30]. In the present study we explored the expression of REPS2 mRNA and miR-675-5p by qRT-PCR analysis. As shown in Figure 6B, miR-675-5p was negatively correlated with REPS2 mRNA in the ESCC samples (*r* = −0.670, *P* < 0.001). These results confirmed that overexpressed miR-675-5p repressesed endogenous REPS2 expression.

## DISCUSSION

Recently, it has been revealed that altered expression of miRNAs contribute to the initiation and progression of cancers [32–36]. The overexpression of miR-675 has been found in human colorectal cancer, pancreatic cancer, serous endometrial tumors and endometrial carcinosarcomas [17–19]. In the present study, we firstly found that miR-675-5pwas constantly up-regulated in ESCC tissues and cell lines. And interestingly, miR-675-5p expression was significantly correlated with lymph node metastasis, TNM stage, overall survival and disease-free survival of ESCC. In particular, high expression of miR-675-5p was significantly associated with poor prognosis in advanced stage and lymph node metastasis patients with ESCC, respectively. More importantly, from multivariate analysis, we obtained sufficient evidence to infer that miR-675-5p expression level was an independent prognostic indicator for ESCC patients. From multivariate analysis, we also found that TNM stage and N2 classification was correlated with overall and disease-free survival, but N classification only related to overall survival, not disease-free survival. After careful consideration, we thought that it might be caused by limited sample size. Anyhow, miR-675-5p might serve as a valuable prognosis biomarker for ESCC patients. Furthermore, we found that H19 was overexpressed in ESCC tissues and positively correlated with miR-675-5p, which corresponded with other reseachers’ studies [16, 17].

Given that miR-675-5p was up-regulated in ESCC tissues and cell lines, we speculated that down-regulation of miR-675-5p might suppress the malignant phenotypes of ESCC cells. The results confirmed that miR-675-5p inhibition induced ESCC cells G1 arrest, and reduced ESCC cells proliferation, colony formation, migration and invasion *in vitro*, tumorigenicity and tumor metastasis *in vivo*. Altogether, the oncogenic effects of miR-675-5p on ESCC cells growth and metastasis might contribute to poor prognosis of ESCC patients with higher expression of miR-675-5p. Our findings also suggest that miR-675-5p could potentially be used as a biomarker to clinically predict metastasis, recurrence and survival prognosis for patients with ESCC.

The basic function of miRNAs is to regulate their target genes by direct cleavage of the mRNA and/or by inhibition of protein synthesis, according to the degree of complementarity with the target mRNA 3′-UTR [37, 38]. Recently, it has been demonstrated that miR-675 have several targets in different cancers, such as c-Cbl and Cbl-b in breast cancer [5], GPR55 in lung carcinoma [26], Rb in colorectal cancer [17], CALN1 in gastric cancer [14], Cadherin 11 in melasma [39]. In the present study, we have demonstrated REPS2 to be target of miR-675-5p in ESCC. Furthermore, we have provided the following series of evidence that miR-675-5p induced ESCC cell G1-S transition and promoted tumor growth, proliferation, migration, tumor metastasis and tumorigenicity in part by suppressing REPS2. Firstly, the miR-675-5p level was inversely correlated with REPS2 level in ESCC tissues. Secondly, down-regulation of miR-675-5p significantly increased REPS2 mRNA and protein levels in ESCC cells. Thirdly, overexpression of miR-675-5p decreased the luciferase reporter activity of wild-type 3′-UTR but not mutant 3′-UTR of REPS2. More importantly, knockdown of REPS2 gene could mimic the oncogenic effect of overexpressed miR-675-5p. These data supported REPS2 as a downstream mediator of miR-675-5p functioned in ESCC. In addition, emerging evidences have shown that REPS2 plays important roles in inhibiting cell proliferation, migration of cancer cells [27, 31, 40–42].

REPS2 is initially identified in a yeast two-hybrid screening as a partner of Ral-binding protein 1 (RalBPl) [43], which is a putative effector protein of Ral and possesses the GTPase-activating (GAP) activity for RACl and CDC42 [44]. Activation of the small GTPases RAC1 and CDC42 mediating cell proliferation and migration has been demonstrated in many cancers, like breast cancer, prostate cancer, lung cancer, head and neck squamous cell carcinoma [45–49]. Furthermore, overexpression of REPS2 might result in a strong inhibition of RAC1 and CDC42 signaling pathway [45]. These researches suggest that signals from REPS2 might suppress tumor growth and metastasis through RalBP1/RAC1/CDC42 signaling pathway. In order to investigate whether miR-675-5p exhibit its oncogenic role via regulating RalBP1/RAC1/CDC42 pathway by targeting REPS2 in ESCC, we examined the expression of downstream target genes in REPS2 signaling pathway and found that the expression of RalBP1, RAC1, CDC42, MMP9, MMP2 and CyclinD1 were decreased in ESCC cells that stably down-regulated miR-675-5p. Inversely, interference of REPS2 in miR-675-5p-inhibition EC9706 cells abrogated the effects induced by miR-675-5p down-regulation, which was similar to the function of miR-675-5p overexpression in EC9706 cells. MMP2 and MMP9 are belonging to matrix metalloproteinases family, which are vital in tumor invasion and heterogeneous adhesion [50]. Consist with the previous researches, our data demonstrated that inhibition of REPS2 would activate RalBP1/RAC1/CDC42, increase MMP2/9, thus promote tumor invasion and metastasis [51, 52]. In the present study, we also found that miR-675-5p could regulate Cylin D1, which mediates G1-S phase [53]. Though the explicit mechanism of tumorigenesis of REPS2 in ESCC is unclear, on the basis of our present study, we inferred that REPS2 was closely related to the RalBP1/RAC1/CDC42 signaling pathway through specific bindings with Ralbp1 which possesses the GAP activity for RACl and CDC42. As above study, REPS2 was identified as a target of miR-675-5p. So we speculated that miR-675-5p/REPS2/RalBP1/RAC1/CDC42 signaling pathway was an important molecular pathogenesis of ESCC. Down-regulation or up-regulation of miR-675-5p would increase or decrease REPS2 expression which inhibited or promoted RalBP1′s GTPase-activating activity, resulting in suppressing or facilitating RAC1/CDC42 signaling pathway. Our noted results corresponded with this speculation.

REPS2 is found to be involved in EGF signaling pathway in prostate cancer [28, 29]. Recently, miR-675 is demonstrated to activate EGF signaling pathway in breast cancer [5]. Consequently, we have explored the underlying interaction between REPS2 and EGF pathway in ESCC. We examined several growth factor signaling genes, including EGFR, pEGFR, AKT, pAKT, Erk and pErk by western blotting. Compared to negative control group, the protein expression of these genes were not obviously decreased in EC9706 and EC109 cells with stably down-regulated miR-675-5p (Supplementary Figure S1A). These results indicated that REPS2 was not involved in growth factor signaling pathway in ESCC. In different cancers, miR-675 induces various phenotypes via diverse signaling pathways [5, 6, 26, 39]. As noted in our manuscript, knockdown of miR-675-5p in ESCC cells induced cell G1 arrest, down-regulated CyclinD1 which was correlated with G1-S phase transition and inhibited REPS2/RalBP1/RAC1/CDC42 signaling pathway, which might be responsible for the effect of miR-675-5p on proliferation of ESCC cells.

Tsang et al. infer that there might be an inverse loopback between the expressions of RB and H19/miR-675 in human colorectal cancer [17]. However, Matouk et al. find that there is a positive feedback loop between H19 and Slug mediated by miR-675 which controls E-cadherin expression [54]. We checked the expression of H19 in miR-675-5p-inhibition EC9706 and EC109 cells and negative control cells by qRT-PCR analysis. The expression of H19 was found no significant difference in these groups (Supplementary Figure 1B), indicating that there was no feedback in ESCC in H19/miR-675/REPS2 pathway.

In conclusion, miR-675-5p was up-regulated in ESCC. miR-675-5p possesses the potency to promote ESCC growth and metastasis by regulating REPS2. miR-675-5p/REPS2/RalBP1/RAC1/CDC42 signaling pathway might be an important mechanism of tumorigenesis of ESCC. Therefore, miR-675-5p could play an oncogenic role in ESCC. The identification of miR-675-5p and its target genes REPS2 in ESCC would help in a better understanding of the molecular mechanisms underlying ESCC development, which would provide us a wider perspective on ESCC intervention/prevention and treatment.

## MATERIALS AND METHODS

### Ethical statement

Written informed consent was obtained from all participants, and the study protocol was approved by the ethics committee of Xiangya Hospital, Central South University (CSU). All mouse experiments were approved by the Animal Care and Use Committee and conducted in accordance with the official recommendations of the Care and Use Laboratory Animals of Xiangya Hospital, CSU.

### Clinical samples and cell lines

ESCC, paired normal esophageal tissues (greater than 5 cm away from the tumor margin) were obtained from 60 patients (median age 59.15 years, range 35–75 years) who underwent esophagus resection between January 2010 and March 2012 at Xiangya Hospital, CSU. All patients had no history of previous malignancies, no history of chemotherapy or radiotherapy. All the samples were immediately snap-frozen in liquid nitrogen and stored at −80°C for RNA and protein extraction. All tumors, paired normal tissues were histologically confirmed by two pathologists.

The subjects were followed-up every 3 months during the first postoperative year and at least 6 months afterward for survival and recurrence inquiry until death or until the end of the investigation.

Human ESCC cell lines (TE-1, EC-1, EC109 and EC9706) were obtained from Chinese Academy of Science cell bank (Shanghai, China). A normal human esophageal epithelial cell line (HEEpic) was purchased from the American Type Culture Collection. Cells were cultured and maintained in RPMI 1640 supplemented with 10% fetal bovine serum (FBS), 100 U/ml penicillin and 100 ug/ml streptomycin in a humidified incubator with 5% CO2 at 37°C. All culture materials were purchased from GIBCO, USA.

### miRNA target predictions and luciferase reporter assay

The miRNA sequence was analyzed using miRBase (http://microrna.sanger.ac.uk/sequences/), and miRNA target predictions of the differentially expressed miRNAs were performed by using miRDB, DIANA-MICROT, MICRORNA.ORG and TargetScan.

The pMIR-report luciferase vector was used for the construction of the pMIR-REPS2-Wt or pMIR-REPS2-Mut vectors. The pMIR-REPS2-Mut vector was built with REPS2 that had undergone site-directed mutagenesis of the miR-675-5p target site using the Stratagene Quik-Change site-directed mutagenesis kit (Stratagene, Germany). EC9706cells were transfected in 24-well plates using Lipofectamine 2000 (Invitrogen, USA) transfection reagent. Each well was transfected with either 100 ng pMIR-REPS2-Wt or 100 ng pMIR-REPS2-Mut vectors together with 30 pmol hsa-miR-675-5p or negative control. pRL-TK (Promega, USA) was also transfected as a normalization control. After 48 hof transfection, luciferase activity was measured by the Dual Luciferase Reporter Assay (Promega, USA).

### Quantitative real-time PCR (qRT-PCR)

qRT-PCR was used to detect the expression of miR-675-5p and the expression of primary transcript of REPS2. Total RNA was extracted from the tissues or cells using Trizol reagent (Invitrogen, USA) according to the manufacturer's protocol. For miRNA qRT-PCR, reverse transcription was performed using the miRNA qRT-PCR Detection Kit (GeneCopoeia, USA). U6 snRNA was measured as an internal control. For mRNA detection, reverse transcription was performed according to the protocol provided with the High-Capacity cDNA Reverse Transcription Kit (Invitrogen, USA). β-actin mRNA levels were used for normalization. qRT-PCR was performed with SYBR Green PCR MasterMix according to the manufacturer's instructions using the ABI 7500 real-time PCR system (Applied Biosystems, CA, USA) and accompanying analytical software. The reaction was first denatured at 95°C for 10 min, then 40 cycles at 95°C for 10 s, 60°C for 20 s and followed by 72°C for 10 s. The qRT-PCR results were analyzed and expressed as relative miRNA expression of CT (threshold cycle) value. Levels of miR-675-5p and REPS2 were calculated using the 2^−ΔΔCt^ method with U6 snRNA and β-actin as an endogenous control, respectively. Experiments were repeated at least three times. The mature hsa-miR-675-5p (5′-UGGUGCGGAGAGGGCCCACAGUG-3′) DNA sequence was used as the forward primer, and the 3′-universal primer provided from the miRNA qRT-PCR Detection Kit (GeneCopoeia, USA) as the reverse primer. For mRNA detection, the gene-specific primers were: H19 primer (142 bp), forward 5′-CCGG ACACAAAACCCTCTAGCT-3′, reverse 5′-TGTTCCGAT GGTGTCTTTGATG-3′; REPS2 primer (126 bp), forward 5′-CTGAAGACCAGCAGACACCA-3′, reverse 5′-TTTA GGATCTGGCCCTGTTG-3′; β-actin primer (205 bp), forward 5′-TGACGTGGACATCCGCAAAG-3′, reverse 5′-CTGGAAGGTGGACAGCGAGG-3′.

### Vector construction, lentiviral production

The sequence of hsa-miR-675-5p-inhibition was constructed as follows: (Forward) 5′-TGGTGCGGAGAGG GCCCACAGTG-3′, (Reverse) 5′-CACTGTGGGCCCTC TCCGCACCA-3′. The sequence was amplified and cloned into the pGCsil-GFP vector (GeneChem Co., China) to generate pGCsil-GFP-miR-675-5p-inhibition (named LV-miR-675-5p-inhibition after virus packaging and production). The non-silencing control sequences (Forward: 5′-TTCTCCGAACGTGTCACGT-3′, Reverse: 5′-ACGTG ACACGTTCGGAGAA-3′) was cloned into the pGCsil-GFP vector as negative control (named LV-miR-675-5p-NC after virus packaging and production). The hsa-miR-675-precursor sequence was constructed as follows: (Forward) 5′-ACCGGTGGAGGGCGAAGC-3′, (Reverse) 5′-GAATTCAAAAACTCCTGAGAG-3′. The sequence was amplified and cloned into the pGCsil-GFP vector to generate pGCsil-GFP-miR-675 (named LV-miR-675-5p-precursor after virus packaging and production) and the pGCsil-GFP vector alone as negative control. Virus packaging, production and cell transfection were performed according to the manufacture's protocol. The expression was validated by qRT-PCR.

### Cell Transfection of miRNA

Briefly, cells at approximately 40% confluence in 6-well plates were transfected with lentivirus (GeneChem Co., China) according to the manufacturer's instructions. The culture medium was changed 12 h post transfection and the cells were cultured for an additional 72 h. Then, cells were harvested for further experimentation. The expression of miR-675-5p was confirmed by qRT-PCR.

### MTT assay, cell cycle analysis and colony formation assay

For MTT assay, cells with different treatment or corresponding negative control cells were dispensed in a 96-well plate with 1500 cells per well. Each group consisted of three wells. The cells were incubated for 24 h, 48 h, 72 h, 96 h. Thereafter, the cells were incubated in 50 μl of 0.1 mg/ml solution of 3-[4,5-dimethylthiazol-2-yl]-2,5-diphenyltetrazoliumbromide (MTT) at 37°C for 3 h and then lysed in 150 μl of dimethylsulfoxide at room temperature for 30 min. The absorbance in each well was measured at 490 nm by a microplate reader at 490 nm. The experiments were performed in triplicate.

For cell cycle analysis, cells were seeded in 6-well plates at 2 × 10^5^ per well. 24 h after transfection, cells were fixed in 70% ethanol at 4°C for 24 hours and stained with 50 μg/ml propidium iodide (Keygen, Nanjing, China). The cell cycle distribution was analyzed by flow cytometry (Epics Altra, Beckman Coulter, USA).

For colony formation assay, cells were counted and seeded (800 cells/well) in culture dish (10 cm) (in triplicate). Fresh culture medium was replaced every 3 days. Colonies were counted only if they contained more than 50 cells, and the number of colonies was counted at 14 days after seeding. The cells were stained using Giemsa. The ability of colony formation was calculated by the colony formation number.

### Transwell migration and invasion assays

The migration and invasion assays were carried out using Transwell insert chambers (Corning, USA). At first, cells were cultured in RPMI 1640 to be serum- starved for 24 h to remove the influence of FBS. Then for migration assay, 6 × 10^4^ cells were plated into the upper chamber in serum-free medium in triplicate. Medium containing 5% FBS in the lower chamber served as chemoattractant. After incubation for 18 h at 37°C in a 5% CO_2_ humidified incubator, cells in the upper chambers were removed by wiping with a cotton swab and cells migrated to the lower surface of filter were fixed in 70% ethanol for 30 min and stained with 0.2% crystal violet for 20 min. Cell migration was scored by counting ten random fields per filter under a light microscope. And for invasion assay, 8 × 10^4^ cells were seeded into upper chambers precoated with matrigel (Corning, USA) in serum-free medium in triplicate. Medium with 5% FBS were added to the lower chamber to serve as chemoattractant. After incubation for 24 h at 37°C, non-invading cells on the upper surface of filter were removed with cotton swabs and invading cells that migrated to the lower surface of filter were fixed, stained and scored as described above.

### Small interference RNA (siRNA)

The REPS2-siRNA (named si-REPS2) sequence was constructed as follows: sense 5′-CCAGAACUCUC CUAUAUAUTT-3′. The non-specific control siRNA (named si-NC) sequence was constructed as follows: 5′-UUCUCCGAACGUGUCACGUTT-3′. The si-REPS2 and si-NC were purchased from GenePharma, China. The miR-675-5p-inhibition EC9706 cells at 50 to 60% confluency were transfected with 100 nmol/liter siRNA-REPS2 or their corresponding negative control cells by Lipofectamine 2000 in Opti-Mem (Invitrogen, USA). 6 h post transfection, the culture medium was replaced with RPMI-1640 containing FBS. 48 h after transfection, cells were harvested for analysis. The transfection efficiency of si-REPS2 was confirmed by qRT-PCR and western blotting detection of REPS2 mRNA and protein expression.

### Western blotting analysis

Cultured cells or tissues were harvested and lysed with RIPA lysis buffer (Beyotime, China) for 30 min on ice. After centrifugation at 12,000 g for 20 min, the concentration of proteins was measured using Bradford's reagent (Bio-Rad laboratories, USA). The protein samples were denatured by boiling for 10 min and loaded onto SDS–PAGE gel for electrophoresis. The proteins were transferred onto PVDF membrane (Millipore, USA) which was then incubated in the blocking solution (5% FBS) at room temperature for 1 h. The anti-REPS2 antibody (Abcam, UK) or anti-RalBP1 antibody (Santa Cruz, USA), anti-RAC1 antibody (GeneTex, USA), anti-CDC42 antibody (GeneTex, USA), anti-MMP9 antibody (Proteintech, USA), anti-MMP2 antibody (Proteintech, USA), anti-cyclinD1 antibody (Abcam, UK), anti-EGFR antibody (Abcam, UK), anti-pEGFR antibody (Abcam, UK), anti-AKT antibody (Abcam, UK), anti-pAKT antibody (Abcam, UK), anti-Erk antibody (Abcam, UK) and anti-pErk antibody (Abcam, UK) was added into blocking solution and incubated at 4°C overnight. The membranes were subsequently incubated with HRP-labeled goat anti-rabbit IgG for 1.5 h at room temperature. Protein expression was normalized against GAPDH expression (RD, USA) or β-actin expression (Bioworld, USA). Bands were visualized by employing the BeyoECL Plus DetectionSystem (Beyotime, China) and Bio-Rad Image Lab Software (CA, USA).

### *In vivo* tumorigenicity and tumor metastasis assay

Male nude mice (4–5 weeks) were purchased from the Department of Laboratory Animal, CSU (Changsha, China). All animals were housed at pathogen-free condition. Animal experiments were performed in accordance with current guidelines for the Care and Use of Department of Laboratory Animal in CSU. For xenografts, nude mice were divided into 2 set. One set (named set A) which was classified into 2 groups (*n* = 5 for each) was subcutaneously inoculated into the left flanks with 1 × 10^6^ miR-675-5p-inhibition EC9706 cells or negative control cells, respectively. For the other set (named set B) which was classified into 2 groups (*n* = 5 for each) intravenous injection was performed with 1 × 10^6^ of miR-675-5p-inhibition EC9706 cells or negative control cells, respectively. For set A tumor size was measured every 2 to 5 days using caliper. Tumor volumes (mm^3^) were calculated by using the following standard formula: [length*width^2^/2]. Mice of set A were sacrificed and photographied 28 days post inoculation.

### Bioluminescence imaging

The mice of set B were administered 2 nmol MMPSense 750 FAST (PerkinElmer, USA) per mouse via intravenous injection 35 days post injection. Mice's lung was dissected 6 hours post injection and imaged by measuring bioluminescence with Fluorescence Molecular Tomography system (PerkinElmer, USA). The data was analysed using TrueQuant software (PerkinElmer, USA).

### Histological analyses

The mice's tumors of set A and lungs of set B were fixed in 10% neutral PB-buffered formalin (pH 7.4). The fixed samples were then embedded in paraffin, and five non-sequential serial sections were obtained per animal. The sections were stained with hematoxylin/eosin (HE) staining and analyzed for the presence of tumorigenicity and metastasis.

### Statistical analysis

Statistical analysis was performed using SPSS software (version 16.0, Chicago, IL). Data were expressed as mean ± standard deviation (SD) from at least three separate experiments. The differences between groups were assessed using Student t test when there were only two groups, or analyzed by one-way ANOVA when there were more than two groups. The χ^2^ test or Fisher's exact test was used for statistical analysis of categorical data. A Spearman correlation test was used for analyzing the correlations between miR-675-5p expression level and the target gene REPS2 expression level. Survival curves were constructed using the Kaplan-Meier method and evaluated using the log-rank test. The Cox proportional hazard regression model was used to identify factors that were independently associated with overall survival and disease-free survival. A two-tailed value of *P* < 0.05 was considered statistically significant.

## SUPPLEMENTARY MATERIAL FIGURE


