# Supplementary material for: miR-675-5p enhances tumorigenesis and metastasis of esophageal squamous cell carcinoma by targeting REPS2
Source: Oncotarget. 2016 Apr 23;7(21):30730–47. doi: 10.18632/oncotarget.8950 (PMC5058713; doi:10.18632/oncotarget.8950)
Supplement: Supplementary file 1 [file oncotarget-07-30730-s001.pdf]

miR-675-5p enhances tumorigenesis and metastasis of esophageal squamous cell carcinoma by targeting REPS2

Supplementary Materials

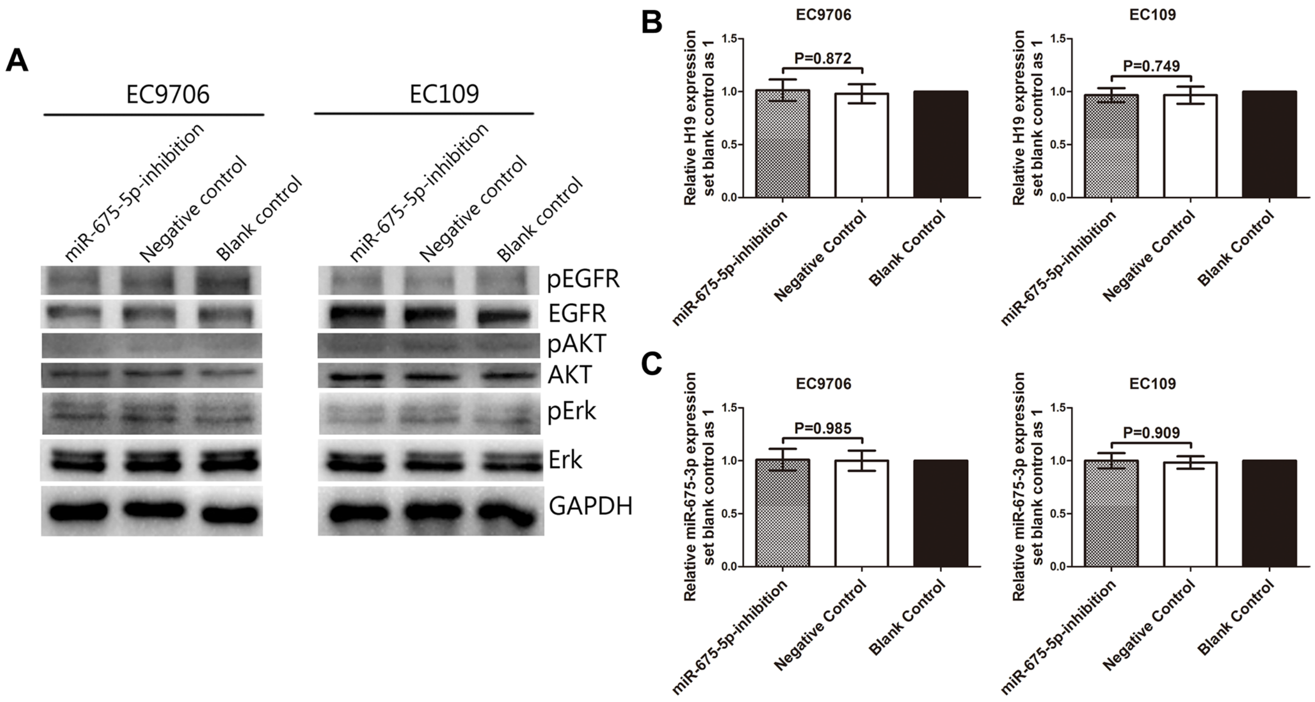

Supplementary Figure S1: miR-675-5p/REPS2 was not involved in growth factor signaling in ESCC and inhibition of miR-675-5p did not affect the expression of H19 and miR-675-3p in ESCC. (**A**) In EC9706 and EC109 cells with miR-675-5p down-regulation, the protein levels of EGFR, pEGFR, AKT, pAKT, Erk and pErk were not obviously decreased compared with negative control (namely cells transfected with LV-miR-675-5p-NC) and blank control group (namely cells without any treatment). (**B**, **C**) The level of H19 and miR-675-3p expression were not significantly down-regulated or up-regulated in miR-675-5p-inhibition EC9706 and EC109 cells.
